# Identification of ADPKD-Related Genes and Pathways in Cells Overexpressing *PKD2*

**DOI:** 10.3390/genes11020122

**Published:** 2020-01-22

**Authors:** Zhe Zhang, Yanna Dang, Zizengceng Wang, Huanan Wang, Yuchun Pan, Jin He

**Affiliations:** 1 Department of Animal Science, College of Animal Sciences, Zhejiang University, Hangzhou 310058, China; zhe_zhang@zju.edu.cn (Z.Z.); 11717011@zju.edu.cn (Y.D.); panyc@zju.edu.cn (Y.P.); 2 Department of Veterinary Medicine, College of Animal Sciences, Zhejiang University, Hangzhou 310058, China; 21817097@zju.edu.cn (Z.W.); whn868@163.com (H.W.)

**Keywords:** ADPKD, RNA-seq, PKD2

## Abstract

Consistent with the gene dosage effect hypothesis, renal cysts can arise in transgenic murine models overexpressing either *PKD1* or *PKD2*, which are causal genes for autosomal dominant polycystic kidney disease (ADPKD). To determine whether *PKD* gene overexpression is a universal mechanism driving cystogenesis or is merely restricted to rodents, other animal models are required. Previously, we failed to observe any renal cysts in a transgenic porcine model of *PKD2* overexpression partially due to epigenetic silencing of the transgene. Thus, to explore the feasibility of porcine models and identify potential genes/pathways affected in ADPKD, LLC-PK1 cells with high *PKD2* expression were generated. mRNA sequencing (RNA-seq) was performed, and *MYC*, *IER3*, and *ADM* were found to be upregulated genes common to the different *PKD2* overexpression cell models. *MYC* is a well-characterized factor contributing to cystogenesis, and *ADM* is a biomarker for chronic kidney disease. Thus, these genes might be indicators of disease progression. Additionally, some ADPKD-associated pathways, e.g., the mitogen-activated protein kinase (MAPK) pathway, were enriched in the cells. Moreover, gene ontology (GO) analysis demonstrated that proliferation, apoptosis, and cell cycle regulation, which are hallmarks of ADPKD, were altered. Therefore, our experiment identified some biomarkers or indicators of ADPKD, indicating that high *PKD2* expression would likely drive cystogenesis in future porcine models.

## 1. Introduction

Autosomal dominant polycystic kidney disease (ADPKD) is one of the most common genetic causes of end-stage renal disease, affecting millions of people worldwide [1]. As a systemic disease, ADPKD is characterized by the formation of gradually enlarged fluid-filled cysts in both kidneys along with extrarenal manifestations such as hypertension, hepatic/pancreatic cysts, intracranial aneurysms, and cardiac valve malformation [2]. Much effort has been made to study the cellular and molecular basis of ADPKD; however, in addition to renal replacement therapy, only tolvaptan has recently been approved for slowing disease progression [3]. *PKD1* and *PKD2* are two causal genes for ADPKD and account for approximately 85% and 15% of cases, respectively [4,5]. The proteins encoded by these genes, polycystin-1 (PC1) and polycystin-2 (PC2), are large transmembrane proteins residing in primary cilia, the plasma membrane, cell junctions, and the endoplasmic reticulum, where they participate in numerous signaling pathways and mediate cell proliferation, Ca^2+^ homeostasis, apoptosis, extracellular matrix deposition, planar cell polarity, and fluid secretion across the epithelium [1,6].

Several genetic hypotheses, e.g., the two-hit [7,8], third hit/renal injury [9,10,11], and dosage effect (reduced or increased *PKD1/2* expression) [12,13], have been proposed to explain the etiology of ADPKD. Of these hypotheses, the two-hit model is widely accepted to explain the focal nature of cystogenesis in the disease, in which somatic inactivation of the remaining normal allele of *PKD1* or *PKD2* gene with the inherited germline mutant allele drives cysts formation. However, exceptional cases were observed, in which persistent expression of polycystins could be noted in cyst lining epithelia of some ADPKD patients, making dosage effect of polycystin above/under certain threshold a plausible explanation of ADPKD [14,15]. In tests of these hypotheses, both *PKD1* and *PKD2* transgenic rodent models exhibited renal cysts and other organ abnormalities [16,17]. Thus, overexpression of either wild-type (WT) or mutant *PKD* genes could drive cystogenesis due to the overexpression dosage effect. However, to date, only murine models have been established, and other animal models are required to recapitulate the phenotype and validate the overexpression model to exclude the species-specific propensity of renal cystogenesis. As pigs, which are similar to human beings in physiology, anatomy, and metabolism, are ideal large animal models for human diseases, we attempted to construct a *PKD2* overexpression porcine model [18]. Unfortunately, no renal cysts were found in these pigs, which exhibited the same phenomena observed in some murine models [19,20]. One possible explanation is that due to low copy numbers or epigenetic silencing, the exogenous transgene is expressed at a level insufficient to drive pathological changes [19,20]. Therefore, to achieve high transgene expression without disruption of endogenous genes, site-specific integration of exogenous gene into safe harbor, would be preferable in future pig studies [21].

In the current study, to explore the feasibility of constructing *PKD2* overexpression porcine models and identifying potential disrupted genes/pathways, LLC-PK1 cells were transfected with four *PKD2* constructs of different genotypes. Via mRNA sequencing (RNA-seq) and enrichment analysis, three commonly upregulated genes (*MYC*, *ADM*, and *IER3*), along with enrichment of MAPK pathways and several biological processes involved in proliferation, apoptosis, and cell cycle regulation, were identified in our cellular overexpression models, indicating that these cellular models likely recapitulate ADPKD. The results not only provide functional information, e.g., biomarkers and disrupted pathways, regarding the progression of ADPKD but also facilitate the future characterization of porcine models of ADPKD.

## 2. Materials and Methods

### 2.1. Plasmids

The plasmid pCAG-hPKD2-3×FLAG containing the WT human *PKD2* gene sequence was customized and purchased from Cyagen (Suzhou, China). Then, a primer pair (PKD2-up and PKD2-down) was used to amplify the entire PKD2-3×FLAG sequence using Phusion high-fidelity polymerase (Thermo Fisher, Shanghai, China). The amplified fragment was gel purified (Thermo Fisher, Shanghai, China) and ligated into the NotI-digested pCAG-floxP-neo-pH11 plasmid using the In-fusion method (Takara, Dalian, China) to construct the pCAG-WThPKD2-3×FLAG-floxP-neo-pH11 (WT) plasmid. Finally, the mutant *PKD2* plasmids pCAG-muhPKD2 c.A1532T/p.D511V-3×FLAG-floxP-neo-pH11 (Tg1), pCAG-muhPKD2 c.T1967G/p.L656W-3×FLAG-floxP-neo-pH11 (Tg2), and pCAG-muhPKD2 c.C2224T/p.R742X-3×FLAG-floxP-neo-pH11 (Tg3) were constructed using a site-specific mutation kit (Beyotime, Shanghai, China). A diagram of the *PKD2* overexpression plasmid is shown in Supplementary Materials Figure S1.

### 2.2. Cell Culture and Transfection

LLC-PK1 cells were obtained from the American Type Culture Collection (ATCC, Manassas, VA, USA) and cultured in 6-well plates in M199 medium (HyClone, GE, Beijing, China) containing 3% fetal bovine serum (HyClone, GE, Beijing, China). When the cells reached 80% confluency, three micrograms of each *PKD2*-containing plasmid and empty plasmid (pCAG-floxP-neo-pH11, NC) were transfected in quadruplicate using Lipofectamine 3000 (Thermo Fisher, Shanghai, China) according to the guidelines. Forty-eight hours later, one of the quadruplicate samples was used for protein extraction, while the other three replicates were used for RNA isolation.

### 2.3. Western Blot Analysis

Protein extraction was performed using Western and IP Cell Lysis Buffer (Beyotime, Shanghai, China). Approximately 30 µg of protein extract was loaded for SDS-PAGE. Anti-FLAG (M2, Sigma-Aldrich, Shanghai, China) was used for immunoblotting at a dilution of 1:1000. Ponceau staining was used as loading control according to the manufacturer’s guideline (Beyotime, Shanghai, China). For detecting the expression of Polycystin-2 and MYC levels in transgenic pigs, kidney proteins from five years old transgenic pigs were prepared using the Western and IP Cell Lysis Buffer (Beyotime, Shanghai, China) according to previously published protocols [18,22]. Anti-PC2 (E20, Santa Cruz Biotechnology, Dallas, TX, USA) and anti-cMyc (9E10, Santa Cruz Biotechnology, Dallas, TX, USA) were employed with dilutions of 1:1000.

### 2.4. RNA-Seq

Forty-eight hours after transfection, cells were harvested, and total RNA was extracted using a MiniBEST Universal RNA extraction kit (Takara, Dalian, China). Then, RNA was subjected to library construction and sequencing on the Illumina HiSeq X Ten platform (San Diego, CA, USA), and quality control was performed according to the Novogene (Beijing, China) pipeline. Salmon software (version 0.14.1) [23], which can efficiently quantify the transcript abundance without aligning reads to a reference genome, was used to quantify transcript-level abundances with a target pig transcriptome as the reference genome (ftp://ftp.ensembl.org/pub/release-98/fasta/sus_scrofa/cdna/). Identification of the differentially expressed gene (DEG) set (adjusted *p*-value ≤ 0.05 and |log_2_Fold Change| ≥ 0.26) was then performed with the DESeq2 (version 1.26.0) package [24]. In addition, a threshold counts per million (CPM) value was set to exclude hits with low expression levels (CPM < 2) [25,26]. The expression data were submitted to the Gene Expression Omnibus with the following accession number: GSE141355.

### 2.5. Enrichment Analysis

DEGs were submitted to the Database for Annotation, Visualization and Integrated Discovery (DAVID) tool (https://david.ncifcrf.gov) to identify significantly enriched gene ontology (GO) terms and Kyoto Encyclopedia of Genes and Genomes (KEGG) pathways [27]. GO terms or KEGG pathways with a *p*-value of ≤ 0.05 were identified as functionally enriched.

### 2.6. Quantitative Real-Time PCR (qRT-PCR)

qRT-PCR was performed using SYBR Green (Takara, Dalian, China) according to a previously established protocol [18]. The primers used are listed in Supplementary Materials Table S1. In brief, one microgram of RNA was reverse transcribed to cDNA (complementary DNA) using an M-MLV kit (Promega, Madison, WI, USA). Then, reactions were performed in a 15 µL volume containing 7.5 µL of SYBR Green, 0.2 µM each primer, and 1 µL of cDNA. The 2^−ΔΔCt^ method was employed to analyze qRT-PCR data [28].

### 2.7. Statistics

One-way ANOVA with Dunnett’s post hoc test was employed to compare the expression levels of human *PKD2*, pig *PKD2*, *MYC*, *ADM*, *IER3*, and *PKD1*. Data are presented as the means ± standard errors of the mean (SEMs). A *p*-value of less than 0.05 was considered to indicate significance.

### 2.8. Ethical Statement

All the procedures were conducted according to the guidelines developed by the China Council on Animal Care and Protocol, and approved by the Animal Welfare Committee of Zhejiang University (no. 11834 issued on 26 February 2018). The experiments conducted on the transgenic pigs were also approved by the China Agricultural University (no. AW31059102-3 issued on 31 March 2019, and no. SKLAB-2012-04-03 issued on 3 April 2012), where these transgenic pigs were.

## 3. Results and Discussion

Currently, no clinically approved drug can halt or reverse ADPKD. Tolvaptan, which is the only available chemical to slow disease progression, targets and inhibits arginine vasopressin V2 receptor mediated cyclic adenosine monophosphate (cAMP) signaling pathways in collecting duct epithelial cells [3]. Since cysts arise from all nephron segments and collecting ducts, cell lines of proximal tubular origin could provide additional useful information regarding ADPKD [29]. In our study, LLC-PK1 cells were used to express four different genotypes of the *PKD2* gene. This cell line was selected because it was isolated from the renal proximal tubule epithelium of a male Hampshire pig and is thus an ideal model for both kidney research and pig biomedical studies [30].

WT *PKD2* was used to establish the pure overexpression model of ADPKD, and the *Tg1*, *Tg2*, and *Tg3* mutations were selected based on the information in the ADPKD mutation database (http://pkdb.mayo.edu) [31]. *Tg1* and *Tg3* are listed as definitely pathogenic; *Tg1* is considered a dominant-negative mutant disrupting intracellular calcium release [32], while *Tg3* lacks the C-terminal tail and cannot interact with PC1 [33]. *Tg2* was identified in a patient with early-onset ADPKD who harbored a homozygous *PKD2-L656W* mutation due to uniparental disomy [34]. The results of the structural study suggested that the *Tg2* mutant is likely pathogenic owing to the disruption of the channel pore structure [35]. The backbone of the transgenic vectors comprises LoxP flanked Neomycin resistant cassette, CAG promoter, and a pH11 (pig Hipp11) sequence (Supplementary Materials Figure S1). The pH11 locus is validated as a promising safe-harbor for overexpressing exogenous genes in pigs [21]. Thus, these plasmids have dual purposes: when using transient expression method, the CAG promoter can drive high expression of transgene; when co-transfection with CRISPR/Cas9 targeting pH11 locus of pig embryonic fibroblasts, the stable transgenic cells could be selected and transgenic pigs can be generated then using marker-free strategy. Both purposes could in principle, maintain relatively high and consistent expression of the transgene.

**Figure 1:**
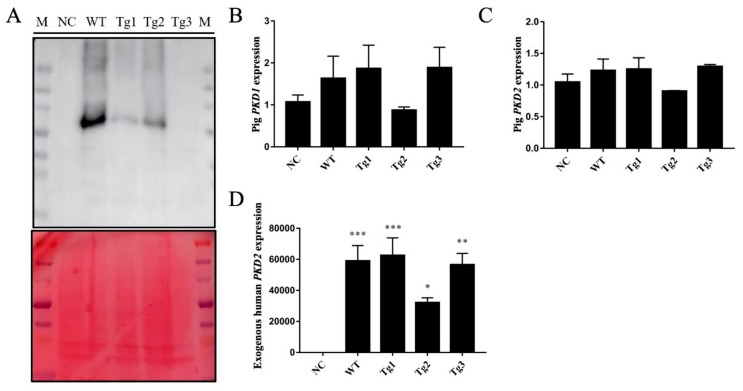
Validation of *PKD2* overexpression in LLC-PK1 cells. (**A**) Western blot analysis of protein extracts from LLC-PK1 cells transfected with different plasmids (NC: pCAG-floxP-neo-pH11, WT: pCAG-WThPKD2-3×FLAG-floxP-neo-pH11, Tg1: pCAG-muhPKD2 c.A1532T/p.D511V-3×FLAG-floxP-neo-pH11, Tg2: pCAG-muhPKD2 c.T1967G/p.L656W-3 × FLAG-floxP-neo-pH11, Tg3: pCAG-muhPKD2 c.C2224T/p.R742X-3×FLAG-floxP-neo-pH11), showing that human *PKD2* was expressed in WT-, Tg1-, and Tg2-transfected cells. Ponceau staining was used in the lower panel as loading control. (**B**–**D**) qRT-PCR results confirmed that endogenous pig *PKD1* (**B**) and *PKD2* (**c**) were not affected by the overexpression of the human *PKD2* gene (**D**). Data are presented as the means ± SEMs. (n = 3; * *p* < 0.05, ** *p* < 0.01, *** *p* < 0.001).

**Figure 2:**
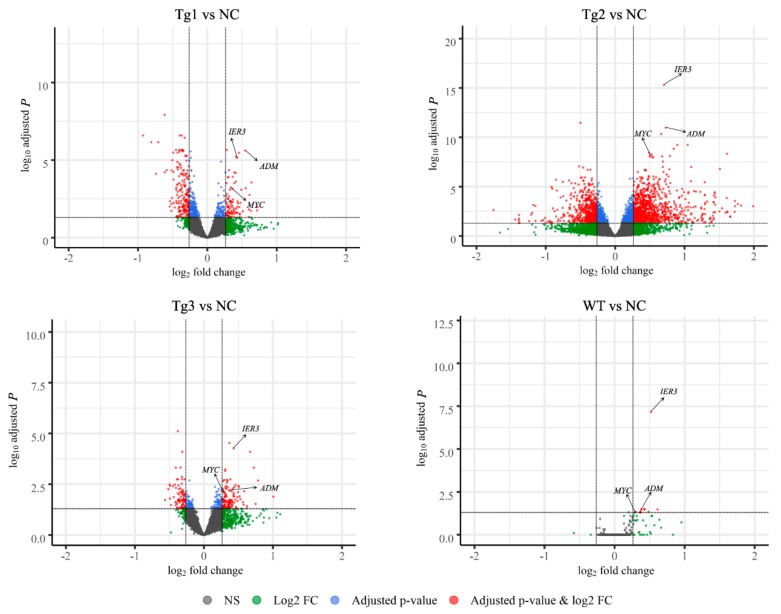
Volcano plots showing the adjusted P-values and the log2 fold change (FC) values of genes in the four genotype models (WT, Tg1-3) versus the control (NC) model. DEGs, indicated by the red dots, were identified as genes with an adjusted *p*-value of ≤ 0.05 and |log2 FC| > 0.26. *MYC*, *IER3*, and *ADM* are indicated by the arrows.

**Figure 3:**
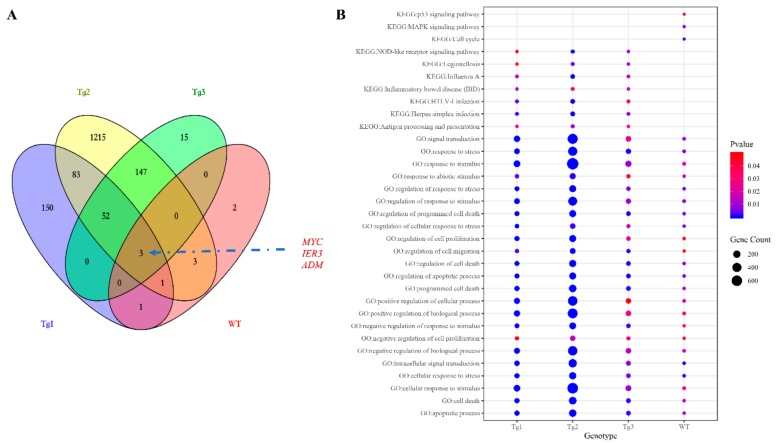
Identification of the shared affected differentially expressed genes (DEGs) and pathways. (**A**) The Venn diagram of DEGs shows that three genes (*MYC*, *IER3,* and *ADM*) were shared by all the genotypes. (**B**) Bubble plot of significant overlapping gene ontology (GO) (biological process) terms enriched in genes from the DEG sets of the four genotypes, significant overlapping KEGG pathways overrepresented in the Tg1-3 models, and KEGG pathways significantly enriched in the wild type (WT) model.

**Figure 4:**
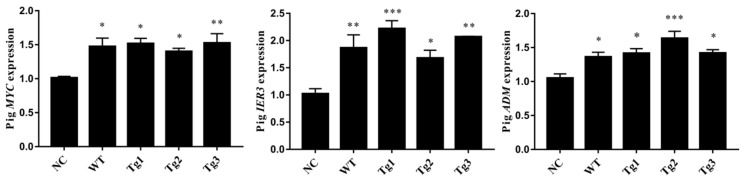
qRT-PCR validation of the shared upregulated DEGs. (n = 3; * *p* < 0.05, ** *p* < 0.01, *** *p* < 0.001).

After transfection of LLC-PK1 cells with the five plasmids, Western blotting was performed to confirm the expression of these proteins except Tg3, which lacks the C-terminal region and the FLAG epitope (Figure 1a). qRT-PCR was then carried out to validate the expression of human *PKD2*. As shown in Figure 1, human *PKD2* had a very high expression level compared to NC, while the endogenous pig *PKD* genes were not affected. As we assumed that overexpression of these *PKD2* constructs affects several signaling pathways and biological processes, some commonly regulated genes and pathways might be identified as potential contributors to ADPKD. Thus, RNA-seq was carried out, and DEGs were identified by separately comparing the reads from the WT, Tg1, Tg2, and Tg3 models with those from the NC model (Figure 2). Ten upregulated DEGs were found in the WT model (Supplementary Materials Table S2). A total of 290 DEGs were found in the Tg1 model; 196 were downregulated, and 94 were upregulated (Supplementary Materials Table S3). In the Tg2 model, 1504 DEGs were observed, comprising 520 downregulated and 975 upregulated genes (Supplementary Materials Table S4). The Tg3 model exhibited 217 DEGs; 89 downregulated genes and 128 upregulated genes (Supplementary Materials Table S5). Among these DEGs, *MYC*, *ADM*, and *IER3* upregulations were found to be common to all four genotypes (Figure 3a), and this finding was further validated using qRT-PCR (Figure 4). The proto-oncogene *MYC* has been well studied as a critical factor in the progression of cystogenesis, and increased *MYC* expression levels have been found in both human patient specimens and murine models of ADPKD [36,37]. In addition, in several transgenic murine models with overexpression of either WT or mutant *PKD* genes, *cMyc* exhibited significant overexpression [38,39,40,41]. Recently, a causal relation between *MYC* and ADPKD has been proposed: in the context of either *PKD1* overexpression or downregulation, *MYC* knockout could ameliorate cystogenesis, suggesting that *MYC* is an essential factor for cystogenesis [42]. In our previous study, *PKD2* transgenic pigs were established based on the overexpression dosage effect hypothesis. The initial tests showed robust expression of exogenous gene in young transgenic pigs, for which we mistakenly believed that renal cysts would form after several years [18]. However, no renal cysts or other morphological manifestations were found later in the pigs. At the molecular level, equal amount of PC2 or MYC levels were noticed in the aging pigs despite the genotypes (Supplementary Materials Figure S2), indicating possible transgene silencing. The construction of the transgenic pigs took advantage of random integration and the CMV promoter, which were reported to be susceptible to epigenetic silencing [43,44,45,46,47,48]. Thus, the findings from our cellular models and transgenic pig model indicate the importance of consistent expression of *MYC* in the context of *PKD2* overexpression, which might lead to cystogenesis in vivo. To our knowledge, no study has focused on the correlation between ADPKD and the *ADM/IER3* genes. However, *MYC/ADM/IER*3 frequently appear in the lists of upregulated DEGs identified via transcriptomic analyses in various ADPKD-related studies [25,26,49,50,51,52,53]. In addition, adrenomedullin, encoded by *ADM*, is a potent hypotensive peptide and has been associated with hypertension and chronic kidney disease (CKD) [54,55]. One study showed that *IER3* was upregulated in a model of CKD [56]. Since ADPKD can be considered a CKD and hypertension is a hallmark of ADPKD, *ADM* and *IER3* upregulation might be useful as indicators of disease progression.

Next, the DEGs identified in these cells were subjected to GO and KEGG enrichment analyses using the DAVID tool (Supplementary Materials Tables S6–S13). The overlapping GO Biological Process (GO_BP) terms shared by the four genotypes (Figure 3b) included cell proliferation, apoptosis, response to stress, response to the extracellular stimulus, response to wounding, cell migration, and cell cycle regulation, which are key features of ADPKD and were also be found in two transcriptomic meta-analyses of ADPKD tissues [50,57]. In the analysis of overexpressed pathogenic *PKD2*, additional terms such as reactive oxygen species, morphogenesis of epithelium, nitric oxygen biosynthesis, MAPK regulation, and cytokine-mediated pathways were also enriched. The 10 DEGs overexpressed in the WT model were predicted to be involved in the MAPK, JNK, and p38MAPK pathways (Supplementary Materials Table S6 and Figure 3b). Thus, MAPK-related pathways were possibly perturbed in our cellular models. MAPK has been extensively studied in cystogenesis; while activated, phosphorylated ERK can enter the nucleus and subsequently mediate the activation of genes involved in abnormal proliferation, ultimately resulting in cyst formation [58]. Other related pathways critical in ADPKD but not commonly shared among the four genotypes included cell adhesion, PI3K pathway, and epidermal growth factor in the Tg1 model; Wnt-associated planar cell polarity, cell adhesion, response to cAMP, Wnt-calcium modulating pathway, and canonical Wnt signaling in the Tg2 model; and response to cAMP in the Tg3 model. KEGG pathways were also studied; however, no enriched term was common to the cellular models. A detailed analysis of each genotype showed that the NOD-like receptor signaling pathway and TNF signaling pathway were shared by the Tg1, Tg2, and Tg3 models. Previous reports suggested that in a diabetic nephropathy model, the NOD2-like receptor signaling pathway promotes renal injury, which is regarded as the third hit in ADPKD [59]. In addition, direct treatment of *PKD2^+/-^* kidneys with TNF-α led to aggravated cyst formation [60]. Thus, both of these pathways might be crucial for cystogenesis. Other pathways were also significantly enriched, e.g., the p53 pathway in the WT model; the JAK-STAT, p53, and NF-kappa B pathways in the Tg2 model; and the HIF-1 pathway in the Tg1 model. Therefore, in our enrichment study in cellular models, many previously characterized signaling pathways involved in ADPKD were identified as potential contributors to cystogenesis.

## 4. Conclusions

In the current study, by employing a cellular *PKD2* overexpression strategy, we identified several disrupted pathways/processes related to ADPKD. Additionally, *MYC*, *ADM*, and *IER3* were identified as potential disease contributors or indicators. This information not only provides the first identification of the shared genes and pathways affected based on the overexpression hypothesis in ADPKD but also lays a foundation for future characterization of porcine models of *PKD2* overexpression.

## Abbreviations:

ADPKDAutosomal dominant polycystic kidney diseasePKD1/PKD2Polycystic kidney disease gene 1/2PC1/2Polycystin-1/2DEGDifferentially expressed geneGOGene ontology; KEGGKyotoEncyclopedia of Genes and GenomesWTWild typeTgTransgenicRNA-seqmRNA sequencingMAPKMitogen-activated protein kinasecDNAComplementary deoxyribonucleic acidcAMPCyclic adenosin monophosphate

## Supplementary Materials

The following are available online at https://www.mdpi.com/2073-4425/11/2/122/s1, Figure S1. Representative schematic of the *PKD2* overexpression vector. A. The *PKD2* overexpression vector can be readily used as a targeted integration transgenic vector when cotransfected with a CRISPR/Cas9 vector targeting the pig H11 locus. Three point mutations (c.A1532T/p.D511V, c.T1967G/p.L656W, and c.C2224T/p.R742X) for Tg1-3, respectively, are shown in solid blue lines above *hPKD2*. B. Representative alignment results of WT, Tg1, Tg2, and Tg3 *PKD2* sequences. Mismatched sites are highlighted in yellow. Figure S2. Western blot analysis of PC2 (left panel) and MYC (right panel) expression in aging transgenic pigs showing similar expression levels of the proteins. Tg-P, the cloned pig with positive integration of exogenous gene; Tg-N, the cloned pig with negative integration of pig *PKD2*. Red arrows indicate the location of corresponding bands of PC2 and MYC. Figure S3. MA plots comparing the four genotype models with the control model. The blue nodes represent DEGs, while the green nodes represent genes that had *p*-values of less than 0.05 but did not satisfy the FC cutoff criterion. The triangles represent values that exceed the axis ranges of the graph. The node size varies with the magnitude of the |FC| value, Table S1. Oligos used in the study, Tables S2–S5. DEGs between the four different genotype models and the control model, Tables S6–S9. GO terms significantly enriched in DEGs of the four different genotype models, Tables S10–S13. KEGG pathways significantly enriched in DEGs of the four different genotype models. Table S14. Expression profile (calculated by DESeq2 in TPM) of all genes across all different genotypes.
