# Supplementary figures and images for: Identification of ADPKD-Related Genes and Pathways in Cells Overexpressing PKD2
Source: Genes (Basel). 2020 Jan 22;11(2):122. doi: 10.3390/genes11020122 (PMC7074416; doi:10.3390/genes11020122)

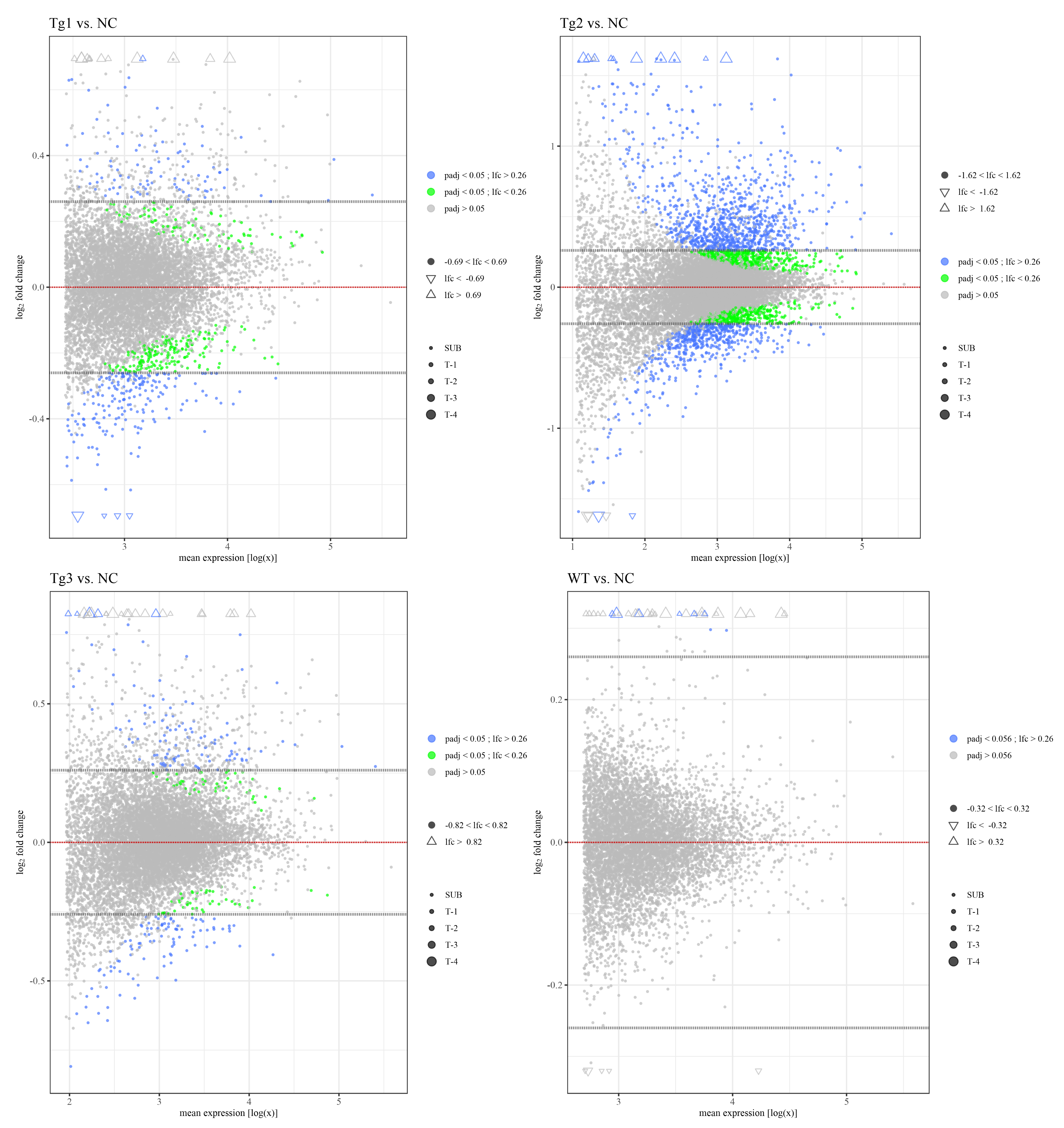

Supplement: Supplementary file 1 [file genes-11-00122-s001.zip › Figure S3.png]

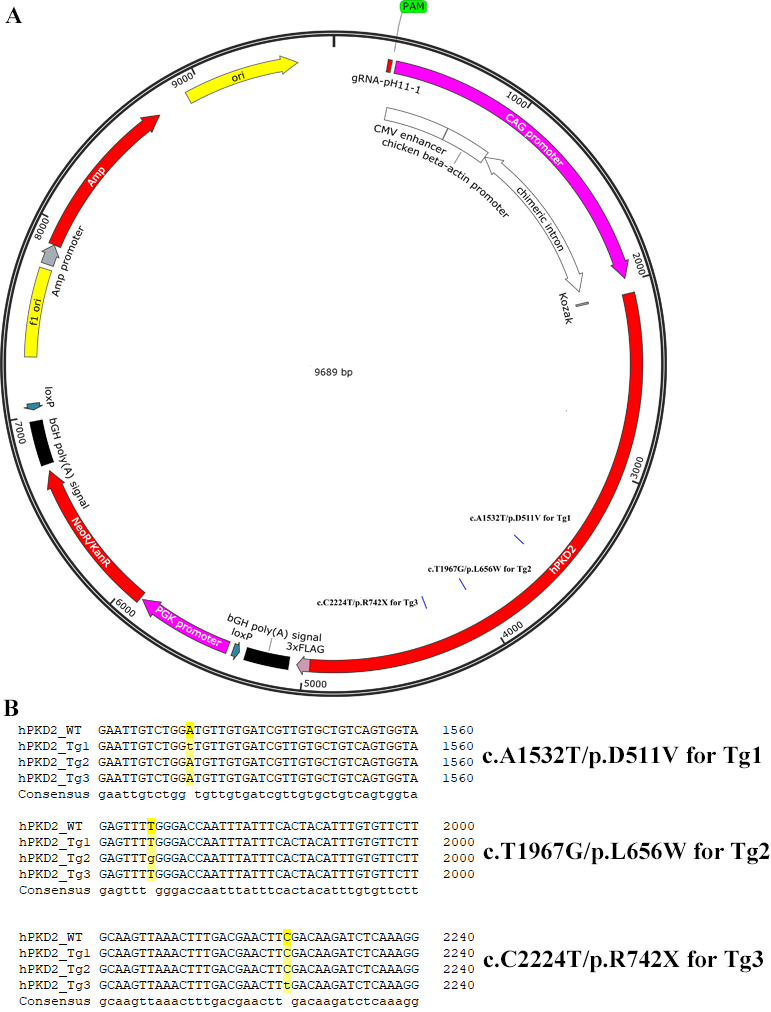

Supplement: Supplementary file 1 [file genes-11-00122-s001.zip › Figure S1.tif]

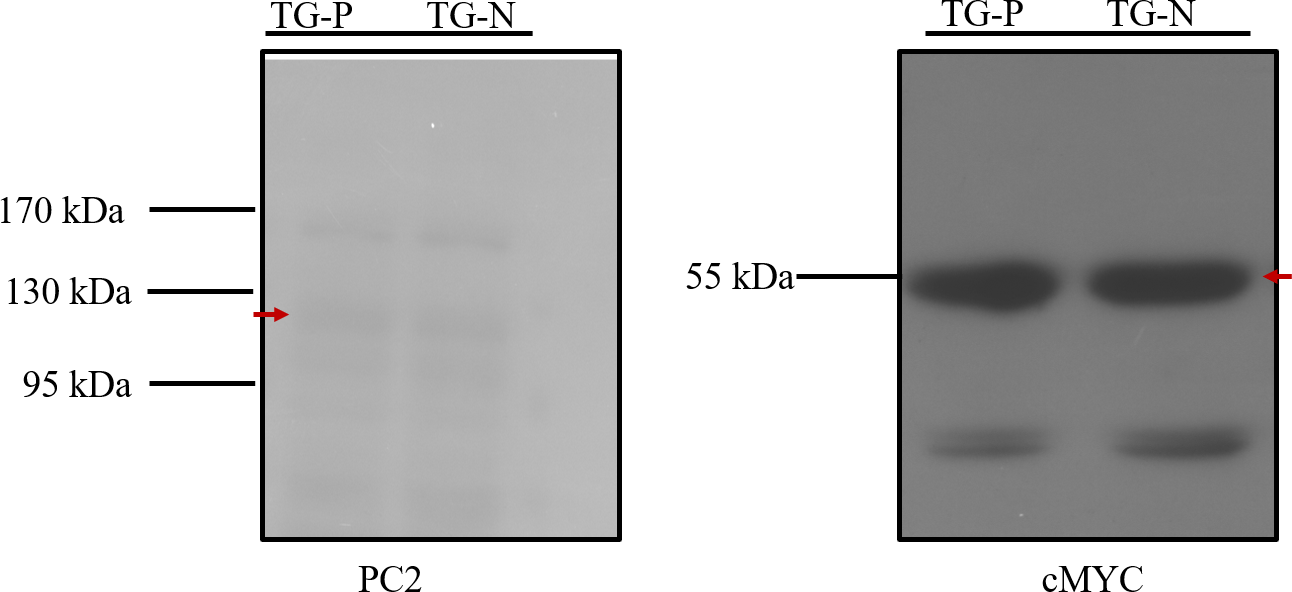

Supplement: Supplementary file 1 [file genes-11-00122-s001.zip › Figure S2.tif]
